# Peptide immobilisation on porous silicon surface for metal ions detection

**DOI:** 10.1186/1556-276X-6-412

**Published:** 2011-06-06

**Authors:** Sabrina S Sam, Jean-Noël JN Chazalviel, Anne Chantal AC Gouget-Laemmel, François F Ozanam, Arnaud A Etcheberry, Nour-eddine N Gabouze

**Affiliations:** 1 UDTS, 2 bd Frantz Fanon, BP 140, Alger-7 Merveilles, Algiers, Algeria; 2 Physique de la Matière Condensée, École Polytechnique, CNRS, 91128 Palaiseau, France; 3 Institut Lavoisier de Versailles, UMR CNRS 8180, Versailles, France

## Abstract

In this work, a Glycyl-Histidyl-Glycyl-Histidine (GlyHisGlyHis) peptide is covalently anchored to the porous silicon PSi surface using a multi-step reaction scheme compatible with the mild conditions required for preserving the probe activity. In a first step, alkene precursors are grafted onto the hydrogenated PSi surface using the hydrosilylation route, allowing for the formation of a carboxyl-terminated monolayer which is activated by reaction with *N*-hydroxysuccinimide in the presence of a peptide-coupling carbodiimide *N*-ethyl-*N*'-(3-dimethylaminopropyl)-carbodiimide and subsequently reacted with the amino linker of the peptide to form a covalent amide bond. Infrared spectroscopy (FT-IR) and X-ray photoelectron spectroscopy are used to investigate the different steps of functionalization.

The property of peptides to form stable complexes with metal ions is exploited to achieve metal-ion recognition by the peptide-modified PSi-based biosensor. An electrochemical study of the GlyHisGlyHis-modified PSi electrode is achieved in the presence of copper ions. The recorded cyclic voltammograms show a quasi-irreversible process corresponding to the Cu(II)/Cu(I) couple. The kinetic factors (the heterogeneous rate constant and the transfer coefficient) and the stability constant of the complex formed on the porous silicon surface are determined. These results demonstrate the potential role of peptides grafted on porous silicon in developing strategies for simple and fast detection of metal ions in solution.

## Introduction

The detection and quantification of heavy metals in the environment are of great importance, due to their high toxicity and their lifetime in soil, air and groundwater. The detection techniques already available are very expensive and difficult to implement. Therefore, there is a real need to develop new detection schemes that are rapid, simple, sensitive and low cost. Electrochemical sensors based on modified surfaces with recognition probes meet these criteria for a fast and easy analysis [1,2], and they are likely to be miniaturised to allow the development of detection equipment capable of operating directly on site. These devices could then complete or even replace the existing conventional techniques.

Surface modification by immobilisation of organic molecules is a very important step and search of new methods is constantly developing [3,4]. The molecular structure, the homogeneity of the layer, the surface density, bonds stability and processes reproducibility are parameters that determine the performance of subsequent applications of these modified surfaces and therefore, must be perfectly controlled.

Furthermore, the choice of the appropriate ligands to be immobilised on the electrode surface is a crucial issue. The majority of work in this field involves a tedious synthesis of selective macrocyclic ligands for a target metal [5]. In nature however, metal binding is achieved with high degree of selectivity using peptide motifs [6].

The known works in this area refer to the immobilisation of peptides on a gold electrode [7,8]. However, the peptide ligands were self-assembled on the surface via a moderately strong gold-sulphur bond. These monolayers are kinetically labile when exposed to moderate temperatures, a chemical attack or application of a potential [9]. Covalent attachment of monolayers on silicon surface through the formation of silicon-carbon bond is an attractive route, since it offers the best performances in terms of robustness and can be made reproducible, with a high yield [10]. In this framework, the advances performed in silicon surface chemistry allowed for attaching functional groups upon demand [11,12]. Generally, the surface functionalization requires a multi-step reaction scheme [13,14]. In addition, the use of porous silicon substrates, allowing for an increased surface interaction area, can enhance the detection signal significantly.

Cyclic voltammetry is an efficient method used extensively to study metal ions complexed to electrodes modified by ligands [15,16]. Parallel to experimental investigations, theoretical studies have been developed to predict and interpret the electrochemical behaviour of this new type of electrodes [17,18].

In this work, Glycyl-Histidyl-Glycyl-Histidine (GlyHisGlyHis)-modified PSi was prepared by anchoring the peptide on a carboxyl-terminated PSi surface using *N*-ethyl-*N*'-(3-dimethylaminopropyl)-carbodiimide (EDC)/*N*-hydroxysuccinimide (NHS) coupling agents. Electrochemical behaviour of such prepared electrodes was carried out in the presence of copper ions by means of cyclic voltammetry. Electrochemical parameters were determined as well.

## Experimental

### Materials

Silicon wafers were purchased from Siltronix, Archamps, France. All cleaning and etching reagents were of VLSI grade and supplied by Merck. Other chemicals were purchased from Sigma-Aldrich (Munich, Germany), Acros Organics (Geel, Belgium) or Fluka (Buchs, Switzerland) and were of the highest purity available. The 10 × PBS buffer (pH = 7.4) was obtained from Ambion (Darmstadt, Germany). Ultrapure water (MilliQ Billerica, MA, USA; 18.2 MΩcm) was used for solution preparation and rinses.

### Porous silicon preparation

The silicon samples of 15 × 15 mm^2 ^size were cut from double-side polished (100) oriented p-type silicon wafers boron doped, 0.08-0.12-Ωcm resistivity and were cleaned in 3:1 96% H_2_SO_4_//30% H_2_O_2 _(piranha solution) for 15 min at 100°C and copiously rinsed with MilliQ water. The native oxide was removed by immersing the samples in 50% aqueous HF for 1 min. The hydrogen-terminated surfaces were electrochemically etched in a 1/1 50% HF/absolute ethanol mixture for 30 s at a current density of 80 mAcm^-^^2^. The prepared PSi surface was rinsed with MilliQ water and dried under a nitrogen stream.

### Peptide immobilisation on the PSi

The freshly prepared PSi sample was transferred into a Schlenk tube containing neat undecylenic acid under argon bubbling and allowed to react at 150°C for 16 h. The PSi surface was subsequently rinsed twice for 30 min in an outgassed Schlenk tube containing acetic acid at 75°C and was blown dry under a nitrogen stream. The surface, now bearing acid terminations, was introduced into a Schlenk tube containing a solution mixture of 5 mM EDC and 5 mM NHS and allowed to react under continuous argon bubbling for 90 min in a water bath at 15°C. The resulting succinimidyl-ester-terminated surface (activated surface) was copiously rinsed with water and dried under a nitrogen stream. The activated surface was immersed in an outgassed Schlenk tube containing a solution of 10^-^^4 ^M GlyHisGlyHis peptide in 1 × PBS buffer at pH approximately 7, overnight. The resulting surface was thoroughly rinsed and dried.

### Infrared measurements

The Fourier transform infrared (FT-IR) spectra were recorded using a Bruker (Equinox 55) spectrometer (Ettlingen, Germany) equipped with a deuterated triglycine sulphate detector. The samples were mounted in a purged sample chamber in transmission geometry at normal incidence. All FT-IR spectra were collected with 200 scans in the 900-4,000 cm^-^^1 ^spectral region at 4 cm^-^^1 ^resolution. Background spectra were obtained by using an untreated deoxidised flat silicon wafer mounted in the same geometry.

### X-ray photoelectron spectroscopy

The X-ray photoelectron spectroscopy (XPS) spectra were obtained with a Thermo Electron VG ESCALAB 220i XL spectrometer (Thermo Electron Corporation, Waltham, MA, USA), using an Al Kα_1 _monochromatic X-ray excitation, and providing an overall full width at half-maximum (fwhm) energy resolution of 0.31 eV.

### Electrochemical detection procedure

All glassware was rinsed with 6 M HNO_3_, then thoroughly with MilliQ water to avoid metal ion contamination.

### Copper accumulation

The copper ions were accumulated at the GlyHisGlyHis-modified PSi electrode at open circuit potential by dipping the sample into 10 mL of a stirred aqueous solution of Cu(II) sulphate in acetate buffer (pH = 8) for 15 min. The sample was removed from the solution, thoroughly rinsed with MilliQ water, dried under a nitrogen stream and transferred to the electrochemical cell.

### Electrochemical measurements

The electrochemical measurements were performed with an Autolab potentiostat using a three-electrode electrochemical cell comprising the modified PSi as working electrode, a platinum wire counter electrode and an Hg/Hg_2_SO_4 _reference electrode. The electrolyte was copper-free ammonium acetate at pH 4 adjusted with HCl. The solution was degassed with argon for 15 min prior to data acquisition. Cyclic voltammetry was performed at different sweep rates between -800 and 0 mV.

## Results and discussion

### Infrared characterization of the modified PSi surface

**Figure 1:**
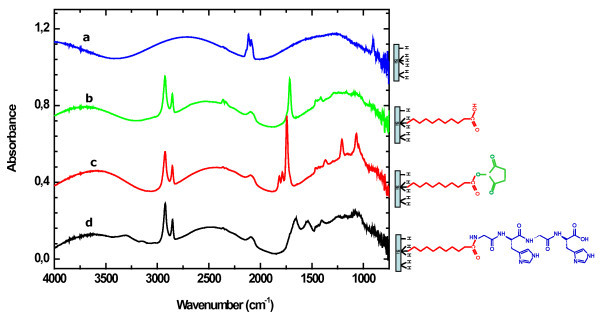
**Transmission IR spectra of modified PSi**. (**a**) Hydrogenated surface after electrochemical fabrication. (**b**) After thermal grafting of undecylenic acid. (**c**) After activation treatment of 90 min in an aqueous solution of 5 mM EDC and 5 mM NHS. (**d**) After amidation in 0.1 mM Gly-His-Gly-His in 1 × PBS buffer.

In the spectrum of the freshly prepared PSi layer (Figure 1a), one observes peaks characteristic of the SiH stretching modes, namely, the bands at 2,085 cm^-^^1^, 2,115 cm^-^^1^and 2,140 cm^-^^1 ^ascribed to monohydride, dihydride and trihydride contributions, respectively [16]. The absence of any sizeable contribution in the 1,000-1,200 cm^-^^1 ^range demonstrates that the PSi surface is oxide free [19]. The peak around 910 cm^-^^1 ^corresponds to the deformation vibrations mode of Si-H_2 _(scissor deformation) [20]. After reaction with undecylenic acid (Figure 1b), the signature of acid chains grafted at the surface appears clearly. It consists of the contribution of the methylene backbone (symmetric and antisymmetric CH_2 _stretching mode at 2,855 and 2,925 cm^-^^1^, respectively, and CH_2 _scissor deformation mode at 1,465 cm^-^^1^), and that of the carboxyl groups (C=O stretching mode at 1,715 cm^-^^1 ^and the C-O-H modes at 1,280 and 1,415 cm^-^^1^) [1,21]. After treatment of the obtained acid surface in EDC/NHS solution [22], a prominent triplet appears (Figure 1c) attesting the formation of a succimimidyl-ester termination. The main peak of this triplet at 1,740 cm^-^^1 ^is ascribed to the antisymmetric stretching mode of the carbonyl groups of the succinimide cycle. The smaller peak at 1,785 cm^-^^1 ^is ascribed to the corresponding symmetric mode, and that at 1,820 cm^-^^1 ^is attributed to the ester C=O stretch [22,23]. Other characteristic bands of the terminal succinimidyl ester group include that with corresponding to the antisymmetric and symmetric stretching of the C-N-C group at 1,205 and 1,370 cm^-^^1^, and the C-O(-N) stretching vibration at 1,065 cm^-^^1 ^[22,23]. After amidation (Figure 1d), the bands corresponding to the terminal succinimidyl ester group disappear and two broad characteristic bands are observed at 1,650 and 1,550 cm^-^^1^, commonly labelled amide I (νC=O) and amide II (δNH) [1,23]. The PSi surface remains essentially oxide free and the SiH stretching band intensities have decreased due to the partial substitution of the surface SiH species by the grafted chains.

### XPS characterization of the modified PSi surface

**Figure 2:**
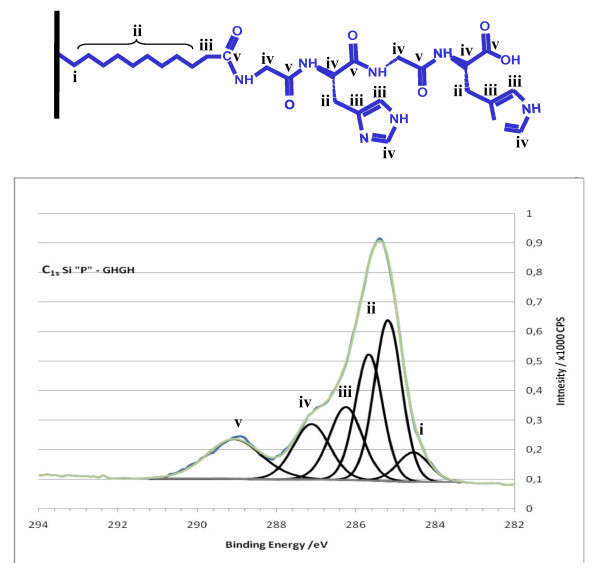
**High-resolution XPS spectrum in the C1s region of GlyHisGlyHis-modified PSi**.

Figure 2 shows the C1s high-resolution XPS spectrum of the GlyHisGlyHis-modified PSi surface. This spectrum shows a peak centred at 285.4 eV with a fwhm of 1.4 eV and a weaker peak at 289 eV. The shoulders observed on either side of the main peak are indicative of the presence of carbon atoms in different environments. The signal can be deconvoluted into six peaks attributed to the different contributions of the carbon atoms. A tentative depiction of the types of carbon atoms that are distinguishable by XPS is shown in Figure 2. The weak peak at 284.5 eV is attributed to the carbon (shown as i) bonded to silicon. The CH_2 _moieties (shown as ii) in the alkyl chain are represented by two peaks. The first one at 285.2 eV is for the nearest carbon atoms from the PSi substrate and the second peak at 285.7 eV is for the carbon atoms close to the attached peptide [24]. The peak at 286.2 eV consists of the carbon atom of the alkyl chain (shown as iii) directly bonded to the peptide and the carbon atoms C=**C**H-N in the imidazole cycles [24,25]. The peak at 287 eV is ascribed to the carbon atoms (shown as iv) adjacent to amide functions and the carbon atoms (N=**C**H-N) in the imidazole cycles. Finally, the contribution at high binding energy (289 eV) is assigned to the acid and amide carbons (shown as v) [24,26].

### Electrochemistry

**Figure 3:**
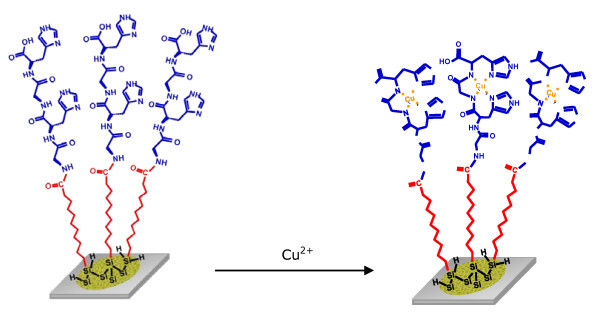
**Reaction scheme of the transition metal complexation on a porous silicon sensor modified with peptide**. In this case, Gly-His-Gly-His chelating Cu(II) cations.

**Figure 4:**
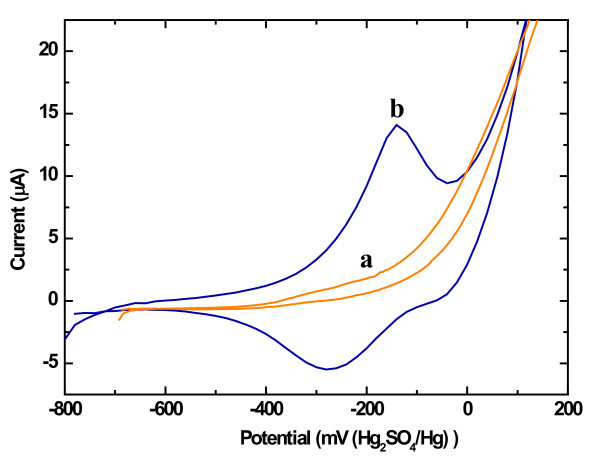
**Cyclic voltammetry of a GlyHisGlyHis-modified PSi surface**. (**a**) Before copper accumulation, (**b**) after copper accumulation. Scan rate = 0.5 V/s.

Figure 3 shows the reaction scheme of copper complexation on the GlyHisGlyHis-modified PSi during the accumulation step. The GlyHisGlyHis-modified PSi electrode is electrochemically inactive (Figure 4a) in the absence of copper (II). After copper accumulation for 15 min in a 0.1 mM Cu^2+ ^solution and washing, the voltammogram recorded in a buffer solution that was free of copper exhibits cathodic and anodic peaks attributed to the quasi-reversible process of the Cu(I)/Cu(II) couple of copper chelated by the GlyHisGlyHis peptide immobilised on the PSi surface (Figure 4b).

### Kinetic parameters determination

Cyclic voltammetry is an efficient method to extract kinetic parameters such as heterogeneous rate constants *k*° and charge transfer coefficients *α *for surface immobilised redox species by examining the variation of peak potential versus experimental time scale (i.e. scan rate). Data analysis relies on a theoretical methodology developed by Laviron et al. [27].

**Figure 5:**
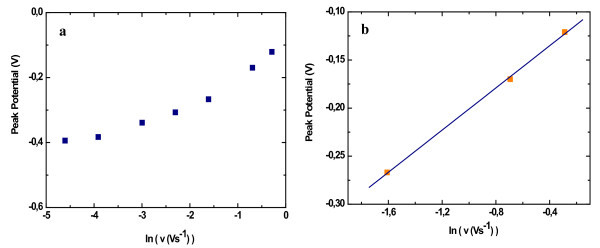
**Plots of the anodic peak potential against logarithm of scan rate for Gly-His-Gly-His-modified PSi after copper accumulation**. (**a**) For all scan rates considered. (**b**) In the case where *E*_p _> 200 mV.

The degree of kinetic reversibility displayed by a surface redox reaction depends on the scan rate. It is expected [28] that a surface redox reaction will exhibit a reversible behaviour (manifested by a peak potential variation quasi-constant with logarithm of scan rate (ln *v*) when the scan rate is small, and an irreversible behaviour (indicated by a linear variation of peak potential with ln *v*) when the scan rate is large. This general prospect was confirmed in our experiments. When the scan rate is higher than 0.02 Vs^-^^1^, the cathodic peak potential *E*_pc _shifts negatively and the anodic peak potential shifts positively with increasing scan rate. Figure 5 shows plots of the anodic peak potential as a function of the logarithm of the scan rate for a GlyHisGlyHis-modified PSi surface after copper accumulation. This figure shows that the peak potentials are practically invariant when the scan rate is low and in contrast for high scan rate, the peak potentials vary linearly as a function of ln *v*.

The heterogeneous scan rate *k*° and the charge transfer coefficient α can be determined using the following equations for the cathodic and anodic peak potentials:(1)(2)

Where *E*° is the standard potential of the surface redox species, *v is *the scan rate (volt/second), *n *is the number of transferred electrons, *R *is the ideal gas constant, *T *is the temperature and *F *is the Faraday constant.

These equations have been established by Laviron considering the limiting conditions where the reaction is totally irreversible. He considered that this case corresponds to the experimental condition where *δE*_p _> 200 mV [27], where *δE*_p _denotes the peak potential separation. In our case, this condition is fulfilled for scan rates above 0.2 Vs^-^^1^. The data *E*_p,a _= *f*(ln *v*) of Figure 5a are replotted as Figure 5b by considering the highest scan rates. The plot yields a straight line with a slope equal to *RT/(1 *- *α)nF *deduced from Eq. 2 and using the anodic potential peak. The value determined for *α *is 0.77.

On the basis of Eqs. 1 and 2, the heterogeneous rate constants *k*° can be calculated with the help of the following expression:(3)

which is valid for *E*_p _> 200 mV. The calculated *k*° value is 1.56 s^-^^1^.

### Apparent stability constant

**Figure 6:**
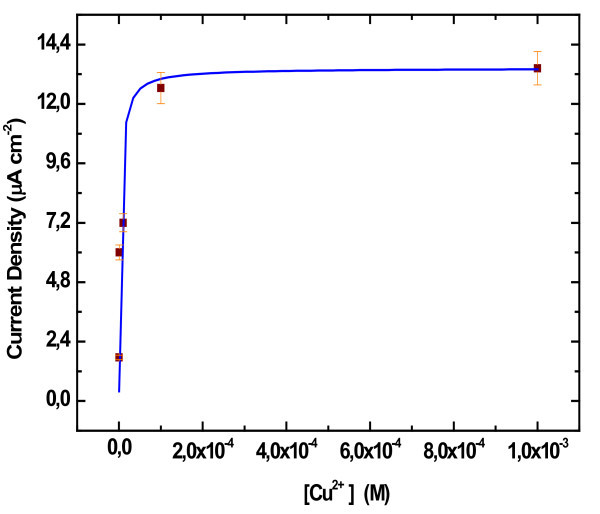
**Calibration curve of anodic peak current density against copper concentration**.

The dependence of the cyclic voltammetry current density at the GlyHisGlyHis-modified PSi on Cu^2+ ^concentration in the accumulation solution was calibrated (Figure 6). Copper ions were accumulated at the GlyHisGlyHis-modified SiP electrodes at open circuit potential by immersing the electrodes into 10 mL of stirred aqueous solutions of copper (II) sulphate of different concentrations (10^-^^7^, 10^-^^6^, 10^-^^5^, 10^-^^4 ^and 10^-^^3 ^M) in acetate buffer (pH = 8) for 20 min. The electrode was then removed, rinsed with copper-free ammonium acetate solution and transferred to a cell with ammonium acetate electrolyte (pH = 4) for cyclic voltammetry.

Figure 6 shows that the relation between current and concentration is clearly non-linear but does follow a "Langmuir" relation. Experimental data for the different Cu^2+ ^concentrations and peak currents were fitted using the following Langmuir equation:

Where *I*_∞ _is the limiting current density corresponding to the saturation of the surface by copper ions, *K *is the pseudo-adsorption coefficient which represents the apparent stability constant of the complex formed on the PSi surface by binding of copper to the GlyHisGlyHis peptide and C is the Cu^2+ ^concentration in the accumulation solution.

The Langmuir curve (solid line in Figure 6) gives a good fit of the experimental data. The value of the limiting current density obtained is 13.44 μA cm^-^^2 ^and the apparent stability constant of the complex Cu-GlyHisGlyHis formed on the PSi surface is *K *= 3 × 10^5 ^M^-^^1^.

The value of *I*_∞ _gives an indication on the sensitivity of the sensor, which has implications for the detection limit whilst the value of *K *is indicative of the affinity of the peptide for the metal ion and hence determines the usable concentration range of the sensor. As a consequence of the high affinity constant for Cu-GlyHisGlyHis, the final sensor is expected to operate in a low concentration range with a low detection limit.

## Conclusion

The GlyHisGlyHis peptide was covalently incorporated onto the PSi structure using multi-step chemistry consisting of: PSi formation, thermal hydrosilylation of undecylenic acid, activation of the acid-terminated surface by formation of a succinimidyl ester, and finally Gly-His-Gly-His anchoring by amidation reaction. Infrared spectroscopy confirmed the efficiency of the process at each stage of surface modification. XPS measurements confirmed the high quality of the grafting and the formation of silicon-carbon covalent bonds. Cyclic voltammetry displayed the ability of the GlyHisGlyHis-modified PSi to complex Cu (II) ions from solution. This result would then demonstrate the role of peptide monolayer in metal detection strategies. The kinetic parameters such as heterogeneous rate constant and transfer coefficient were extracted from the cyclic voltammetry measurements. The apparent stability constant was also determined.

## Competing interests

The authors declare that they have no competing interests.

## Authors' contributions

SS conceived and designed the study, carried out all the experiments and analysis (Porous silicon formation, peptide immobilisation, electrochemical measurements, and Infrared analysis) and drafted the manuscript. JNC participated to the study, to coordination and helped to draft the manuscript. ACGL designed the functionalization part of the study. FO participated to the discussions and coordination. AE performed the XPS analysis. NG participated to the discussions.
